# C2H2 Zinc Finger Proteins Response to Abiotic Stress in Plants

**DOI:** 10.3390/ijms23052730

**Published:** 2022-03-01

**Authors:** Yihua Liu, Ali Raza Khan, Yinbo Gan

**Affiliations:** 1 College of Agriculture and Forestry Sciences, Linyi University, Linyi 276000, China; 2 Zhejiang Key Lab of Crop Germplasm, Department of Agronomy, College of Agriculture and Biotechnology, Zhejiang University, Hangzhou 310058, China; alirazakhan.qau@gmail.com

**Keywords:** C2H2 zinc finger proteins, abiotic stress responses, structure, function

## Abstract

Abiotic stresses have already exhibited the negative effects on crop growth and development, thereby influencing crop quality and yield. Therefore, plants have developed regulatory mechanisms to adopt against such harsh changing environmental conditions. Recent studies have shown that zinc finger protein transcription factors play a crucial role in plant growth and development as well as in stress response. C2H2 zinc finger proteins are one of the best-studied types and have been shown to play diverse roles in the plant abiotic stress responses. However, the C2H2 zinc finger network in plants is complex and needs to be further studied in abiotic stress responses. Here in this review, we mainly focus on recent findings on the regulatory mechanisms, summarize the structural and functional characterization of C2H2 zinc finger proteins, and discuss the C2H2 zinc finger proteins involved in the different signal pathways in plant responses to abiotic stress.

## 1. Introduction

The zinc finger proteins (ZFPs), named from the ‘finger-like’ zinc finger, are one of the largest families of transcription factors and are abundantly distributed in the plant kingdom [1,2,3,4]. ZFPs harbor a highly conserved domain which consists of approximately 20–30 amino acid residues with a consensus sequence of CX2–4CX3FX5LX2HX3–5H (X represents any amino acid, subscript: the number of amino acid) [1,2,5]. The classical zinc finger domain is an important structural motif involved in proteins specific binding to DNA/RNA, protein–protein recognitions and interactions, and membrane association [2,5,6]. It is known that many ZFPs function as a key transcriptional regulator in multiple biological processes of plants, including hormone signal transduction, DNA/RNA combination, transcription regulation, plant growth and development, trichome and root hair development, environmental stress responses [5,6,7]. As diverse as zinc finger proteins’ functions are, their structures are also varied and divided into different classes according to the numbers and positions of the cysteine (Cys) and histidine (His) residues that bind the zinc ion [6]. Based on this canonical classification method of zinc finger proteins, the members of these classes include C2H2 (TFIIIA), C2HC (Retroviral nucleocapsid), C2HC5 (LIM domain), C2C2, C3HC4 (RING finger), C4 (GATA-1), C4HC3 (Requium), C6 (GAL4), and other classes [3,6,8,9]. Among these subclasses, the C2H2 zinc finger proteins contain one of the best-characterized DNA-binding motifs, which are composed of two Cys and two His residues together with one zinc ion tetrahedrally [2,6]. The C2H2 type of zinc finger proteins (C2H2-ZFPs) are well studied and account for a large proportion of the zinc finger protein (ZFP) family. In *Arabidopsis thaliana*, 176 C2H2-ZFPs have been reported, and 189, 109, 321, 118 and 47 C2H2-type ZFPs have been identified in rice (*Oryza sativa*), poplar (*Populus trichocarpa*), soybean (*Glycine max*), tobacco (*Nicotiana tabacum*) and wheat (*Triticum aestivum*), respectively [10,11,12,13,14]. Function analyses showed that C2H2-ZFPs are involved in regulating multiple growth development processes and resisting biotic and abiotic stress in plants [2,3,4,15].

Abiotic stresses, such as drought, high salinity, and low temperature, are one of the serious factors and severe threats to plant growth and development. Huge losses of up to 50% of the total major crops yield are caused by a variety of abiotic stresses [16,17,18,19,20]. In the face of adverse abiotic stresses, plants have evolved stress response mechanisms to respond to stresses and induced adaption to the environment, including morphological and physiological changes [21,22]. These adaptive changes are mostly regulated through activating the signaling transduction pathway, triggering the action of plant transcription factors and controlling the expression of various stress-regulated genes [1,2,5]. C2H2-ZFPs regulate the expression of stress-regulated genes and act as an important part of abiotic stresses response networks [1,2,5]. Recently, increasing studies have demonstrated that C2H2-type zinc finger proteins play important roles in abiotic stress response with a putative repression activity to abiotic stresses in plants [15,22,23,24,25]. In this review, we summarize the important structural features of C2H2 zinc finger proteins, highlight the role of C2H2-ZFPs in plant stress response networks, and conclude the pathway of C2H2-ZFPs in response to abiotic stress.

## 2. Structure and Classification of C2H2-ZFPs

The C2H2-type zinc finger, also known as TFIIIA type or Kruppel-like type finger, is mainly found in eukaryotes [2,6,7,9]. It is reported that each C2H2-type finger has one to four zinc fingers motif(s), which are composed of one zinc ion combined with two Cys and two His residues [6,8,26]. Two Cys and two His form coordination bonds with zinc atoms, which generate a stable finger-like structure [6]. The finger-like structure extends into the large groove of DNA double helix and specific contacts with DNA bases, so that it is capable of playing a role in transcriptional regulation [3,6]. In this finger-like structure, each finger domain contains one α-helix in the C-terminal and two β-strands in its N-terminal, creating a relatively independent structure. The zinc atom is sandwiched between an α-helix and two antiparallel two β-strands, which form a tetrahedral ββα structural system [2,6]. This characteristic ββα structure is stable by interlaced linkage generated by zinc ion, and it can be present in tandem repeats to maintain the structural stability of ZFPs [17,27,28]. In particular, plant-specific C2H2-ZFPs (Q-type C2H2-ZFPs) have a different length of the long spacer between the two zinc fingers in comparison with other eukaryotic organisms. Studies have shown that 64 Q-type C2H2-ZFPs were identified in *Arabidopsis*, as well as 99 and 96 Q-type C2H2-ZFPs in rice and durum wheat, respectively. Q-type C2H2-ZFPs generally include a highly conserved sequence of QALGGH, which confers that zinc finger proteins recognize the target genes and regulate their expression level [2,7,9]. In addition, many Q-type C2H2-ZFPs also have other three characteristics and multifunctional regions [1,5,8,26,28,29]. First, the B-box region, encoding a core sequence KXKRSKRXR, acts as a nuclear localization signal (NLS) in the N-terminal of protein sequences. Secondly, the L-box motif is a short and leucine-rich region between the B-box and the first zinc finger domain. The L-box motif, encoding a core sequence EXEXXAXCLXXL, was also reported to be related to protein interactions. Thirdly, the EAR motif (ethylene-responsive element-binding factor associated with amphiphilic repression domain), also known as DLN-box, is a short hydrophobic part found at the C-terminal region. The hydrophilic and hydrophobic amino acid residues of the EAR motif alternated with aspartic acid residues [8]. The EAR motif, encoding a consensus sequence DLNL, is considered to acquire repression activity, and can regulate the transcription of target genes [6,7,10]. At present, the EAR motif is still the focus of C2H2-type zinc finger protein structure research.

There are many classification types for C2H2 zinc finger proteins following different classification criteria. Generally, C2H2 zinc finger proteins are classified according to the series or dispersion of zinc finger domains, the interval length between zinc fingers, the number of zinc finger domains and the QALGGH sequence [1,5,9]. By those criteria, all C2H2-type ZFPs are divided into three different sets (A, B, and C) in *Arabidopsis*. Each set is further divided into several different subsets (e.g., C1, C2 and C3). The A1 subset and C1 subset, which are reportedly involved in transcriptional regulation, are known as the largest and evolutionarily youngest families among the plant-specific C2H2-type zinc finger proteins [7,13]. The C2 subset and C3 subset are evolutionary older than A1 and C1 subsets and are involved in the ancient cellular pathway [7]. According to the number of zinc-finger domains, the C1 subset is subdivided into five subclasses (C1-1i, C1-2i, C1-3i, C1-4i, and C1-5i) in *Arabidopsis* [2,7,13]. C1-1i indicates the presence of one zinc finger in the ZFP sequence, C1-2i is two zinc fingers, C1-3i is three zinc fingers, and C1-4i is four zinc fingers, whereas C1-5i is five zinc fingers. The C1-2i subclass is the most widely studied and plays a key role in plant development and stress responses.

## 3. Biological Functions of C2H2-Type Zinc Finger Protein

C2H2-type zinc finger proteins play a critical role in many biological functions, including DNA/RNA binding, protein interactions and transcriptional regulation [1]. It was mentioned that the QALGGH motif in the α-helical region is important for DNA binding activity in plants [2]. In animals, the tetrahedral structure, containing a helix and a two-stranded antiparallel sheet enables C2H2-type zinc finger protein to bind the promoters of target genes and regulate their expression [7]. In contrast to the animal, a conserved QALGGH motif positioned in the DNA-binding region of each finger, is essential for sequence-specific DNA recognition and binding [10,30]. In the QALGGH domain, any amino acid mutation will affect its DNA binding ability [5,31,32]. However, the QALGGH sequence is not ubiquitous in every C2H2 zinc finger protein, and it is not the only key site for C2H2 zinc finger protein binding target genes [6]. The long spacers between the two adjacent zinc finger domains were also thought to be related to DNA-binding [33]. In addition to DNA-binding, C2H2 zinc finger proteins can bind to RNA based on their specific bases and specific folding backbones. The diversity of the phosphoric acid skeleton in RNA is necessary for the C2H2 zinc finger protein recognizing and binding RNA specificity sequence [5,34,35]. A phage display technology assay showed that amino acid residues at −1 and +2 positions of the a-helix in C2H2 zinc finger proteins play a decisive role in RNA binding [5]. For protein interactions, C2H2 zinc finger proteins can interact with specific proteins to bind to other DNA sequences or to prevent binding to DNA sequences, in turn, to regulate gene transcription and expression [36,37]. Furthermore, C2H2 zinc finger proteins can recognize similar types of zinc finger proteins or other types of proteins for interaction [5]. Particularly, both the L-box motif and EAR motif of C2H2 zinc finger proteins were identified to be related to protein interactions [38,39]. The EAR motif in C2H2 zinc finger proteins is essential for transcriptional inhibitory activity. Studies have shown that the EAR motif is the smallest known and the first reported repression domain in plants [40,41]. The deletion or mutation of the EAR motif will produce a phenotype similar to the deletion mutant, indicating that this motif is the key domain for the function of the C2H2 zinc finger protein [41]. The EAR motif usually acts as a transcriptional repressor to control the initiation of stress-activated gene expression under various stresses [6]. The majority of stress-related genes were reported that can be regulated by C2H2-type zinc finger proteins with EAR motifs under conditions such as drought, high salinity and extreme temperature [2,5,8]. Moreover, the DNA-binding motif of C2H2-type zinc finger proteins integrate with cis-acting elements in the promoter region of their target genes and regulate their expression level [33]. It was reported the possible binding sequence of C2H2 zinc finger proteins in promoter domains, including A[AG/CT]CNAC, TGCTANNATTG, and TACAAT [5,42,43,44]. However, the complex regulatory mechanism of C2H2-type zinc finger proteins is not yet clear.

## 4. C2H2 Zinc Finger Proteins in Response to Abiotic Stress

**Figure 1:**
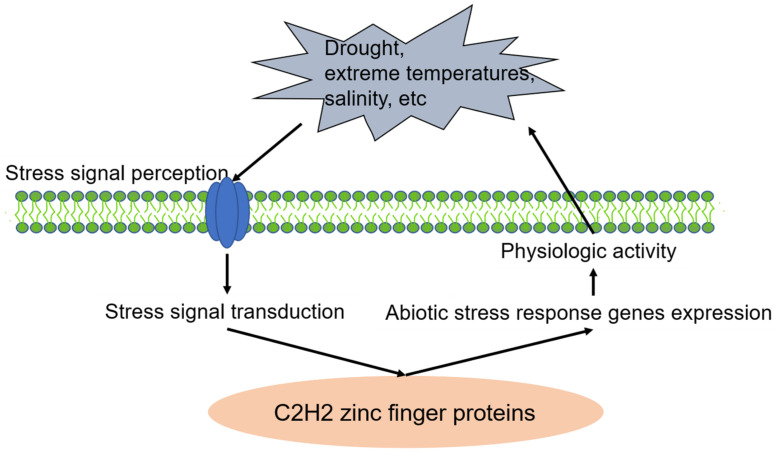
C2H2 zinc finger proteins involved in plant stress responses.

**Figure 2:**
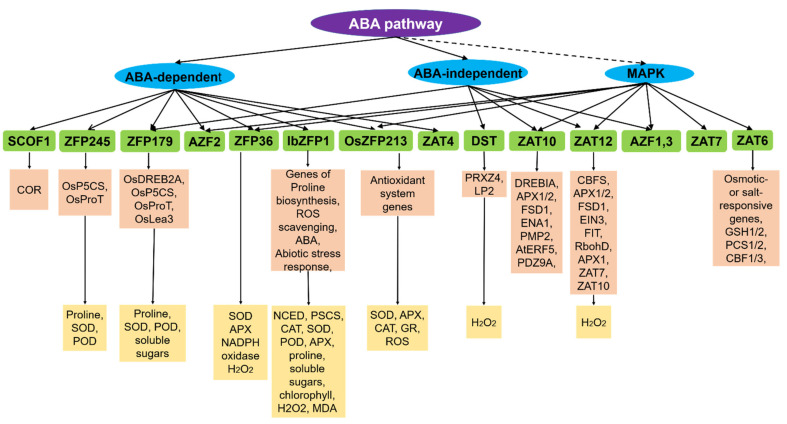
C2H2 zinc finger proteins involved in three signaling pathways response to abiotic stress. The solid lines represent established regulation, and the dashed lines represent putative ones. SCOF-1, ZFP245, ZFP179, AZF1/2/3, ZFP36, IbZFP1, OsZFP213, ZAT4, DST, ZAT10/STZ, ZAT12, ZAT7 and ZAT6 are C2H2 zinc finger proteins. The following abbreviations are used: ABA—abscisic acid; MAPK—mitogen activated protein kinase; COR—cold regulated genes; OsP5CS—pyrroline 5 carboxylate synthetase; OsProT—proline transporter; OsDREB2A—dehydration-responsive element-binding 2A; ROS—reactive oxygen species; PRXZ4—peroxidase 24 precursor; LP2—plasma membrane receptor-like kinase leaf panicle 2; DREB/CBF—C-repeat binding factors; APX—ascorbate peroxidase; FSD1—iron superoxide dismutase 1; ENA1—Na+- exporting P-type ATPase gene; PMR2—Ca^2+^ ATPase gene; AtERF5—a class I ERF protein; RD29A—a classical stress-response gene; EIN3—ethylene-induced 3; FIT—fer-like iron deficiency-induced transcription factor; RbohD—respiratory burst oxidase homologue; GSH1/2—glutathione 1/2; PCS1/2—phytochelatin synthases 1/2; SOD—superoxide dismutase; POD—peroxidase; NCED—9-cis-epoxy-carotenoid dioxygenase; CAT—catalase; MDA—malonaldehyde; GR—glutathione reductase.

C2H2-type zinc finger proteins play an extensive role in plant tolerance to various abiotic stress, such as drought, high salt, cold, high light, osmotic and oxidative stresses [1,5,30]. In the process of external stress resistance, plants have evolved a set of complex and effective defense mechanisms, including signal perception, signal transduction, transcriptional regulation and response, to reduce or avoid damage to plants and ensure their normal growth (Figure 1) [1,5,23,45,46]. Many C2H2-type zinc finger proteins involved in the abiotic stress signaling pathway were identified based on stress induction analysis, mutant analysis, complement analysis and ectopic expression analysis. Phytohormones are responsible for abiotic stress resistance and participate in the process of response to various stresses via C2H2 type zinc finger proteins, especially ABA (Abscisic acid) [47,48,49,50]. ABA, acting as a pivotal regulator of abiotic stress responses in plants, induces the expression of stress-related genes and triggers a range of adaptive physiological responses under abiotic stress conditions in the plant [49,50]. C2H2 zinc finger proteins regulate plant in response to abiotic stresses through two ABA-mediated signal pathways: ABA-dependent and ABA-independent signal pathways [2,48,50,51]. In addition to the ABA signal pathway, C2H2 zinc finger proteins enhance abiotic stress resistance by the MAPK (mitogen-activated protein kinase) signaling pathway [48,51,52,53]. MAPKs, as highly conserved signaling transduction modules, play an essential role in regulating responses to adverse environmental stresses [53]. Typically, a MAPK module is composed of at least three protein kinases, including MAPK (MPK), MAPK kinase (MAPKK/MAP2K/MKK/MEK) and MAPK kinase kinase (MAPKKK/MAP3K/MEKK). The MAPK cascade amplifies and conveys stress signals from signaling receptors to downstream stress response factors through a sequential phosphorylation manner [52,53,54]. Thus, C2H2 zinc finger proteins regulate abiotic stress responses via both the ABA signaling pathway and MAPK signaling transduction pathway and constitute a certain degree of crosstalk and a complex regulatory network (Figure 2).

### 4.1. ABA-Dependent Signal Pathway

C2H2-type zinc finger protein SCOF-1 induced by ABA plays a role in response to cold through the ABA-dependent signaling pathway in soybeans [55]. *SCOF*-1 overexpression transgenic lines significantly increase the tolerance to cold and freezing stress in sweet potato and transgenic potato [56]. Overexpressing *SCOF*-1 in *Arabidopsis* and tobacco activates the transcription of *COR* (cold-regulated genes, such as *COR15a*, *COR47* and *RD29A*) and promote their expression, resulting in enhanced cold tolerance. However, the SCOF-1 protein does not directly interact with CTR/DRE (C-repeat/dehydration element), ABRE (an ABA response element) DNA and cis-acting elements in the promoter regions of *COR* target genes. Interestingly, SCOF-1 interacts with SGBF-1, a soybean bZIP transcription factor and G-box binding factor, to increase the DNA binding to ABRE sequences and promote *COR* genes expression, resulting in enhancing cold tolerance [56]. These results showed that SCOF-1 interacting with SGBFs, regulates COR genes expression and responds to cold stress through an ABA-dependent pathway [55,56,57]. ZFP245 which has a 35% identity in amino acid with SCOF-1, is involved in increasing resistance to various abiotic stresses in rice, including drought, oxidation and cold [58]. ZFP245 exhibits trans-activation activity with a DLN-box/EAR-motif at its C-terminus and a CRT/DRE element in the promoter region. The overexpression of *ZFP245* showed the tolerance of drought and cold stress in rice [58,59]. *ZFP245* is also strongly induced under cold and drought stresses [58]. Under stress conditions, ZFP245 could elevate the expression of pyrroline-5-carboxylate synthetase *OsP5CS* and proline transporter *OsProT* [59]. In addition, the overexpression of *ZFP245* could promote resistance to H_2_O_2_ and increase the content of osmotic adjustment substances, such as free proline, SOD (superoxide dismutase) and POD (peroxidase) in rice [59]. These results indicate that *ZFP245* could improve the tolerance of abiotic stress and oxidation stress through proline biosynthesis and ROS (reactive oxygen species) scavenging. Importantly, overexpressed *ZFP245* could increase plants sensitivity to exogenous ABA, suggesting that ZFP245 is likely to be involved in response to abiotic stress by the ABA dependent signal pathway [59]. Similar to ZFP245, ZFP179 is also isolated from rice and contains a DLN-box/EAR-motif at its C-terminus [60]. ZFP179 could improve salt tolerance through the ROS scavenging system [60,61]. *ZFP179* overexpression in transgenic plants increase the concentrations of proline, soluble sugars, SOD and POD, leading to enhanced salt tolerance [60]. Moreover, overexpression of *ZFP179* showed the upregulating various genes involved in the biosynthesis of osmotic substances, including *OsDREB2A* (*dehydration-responsive element-binding 2A)*, *OsP5CS*, *OsProT* and *OsLea3* [60]. In addition, *ZFP179* could be induced by NaCl, PEG 6000, and ABA treatments in the seedlings, and the overexpression of *ZFP179* showed the high sensitivity of plants to exogenous ABA [60]. Thus, ZFP179 may play a key role in salt stress by activating the expression of *OsP5CS* and *OsProT* through the ROS scavenging system in the ABA-dependent pathway.

AZF (Arabidopsis zinc-finger protein) containing an EAR motif at its C terminus with trans-activation activity suggests that it plays an important role in regulating stress response in *Arabidopsis* [33,62,63]. Moreover, it is reported that AZF specifically recognizes the repeated A[G/C]T core sequences of A (G/C) T-X3~4-A (G/C) T in the promoter regions of stress-related genes, in turn activating or inhibiting their transcription and enhancing plant tolerance to abiotic stress. Among them, *AZF2* is strongly induced by ABA, cold and salt, and probably responds to cold and salt stresses through the ABA-dependent pathway [33,62]. Moreover, *AZF2* contains the ABRE sequence (the ABA-responsive element) in its promoter region. Thus, AZF2 probably regulates the expression of ABA-dependent genes by its repression activity.

Recently, several new C2H2 zinc finger proteins were isolated and identified that play important roles in response to abiotic stresses in the ABA signaling pathway. H_2_O_2_ and ABA-responsive gene *ZFP36* with two C2H2-type zinc finger motifs were shown to participate in water and oxidative stress responses in rice [64]. Under water and oxidative stress, *ZFP36-RNAi* lines showed more sensitivity to H_2_O_2_ and PEG (polyethylene glycol) in contrast with overexpression and WT [65]. Overexpressing *ZFP36* could elevate the expression level and the activities of SOD and APX (ascorbate peroxidase), in turn to enhance the tolerance to water and oxidative stresses, whereas *ZFP36-RNAi* lines exhibit contrary results [65]. Meanwhile, ZFP36 regulated the expression level of NADPH oxidase and the production of H_2_O_2_ in the ABA signaling pathway. Recent results indicated that ZFP36 interact with OsLEA5 (embryogenesis abundant protein) to co-regulate the promoter activity of OsAPX1 in rice [65,66]. IbZFP1 originally isolated from sweet potato is the other important abiotic stress-related C2H2-type zinc finger protein [67]. *IbZFP1* induced by NaCl, PEG and ABA could enhance tolerance to salt and drought in transgenic *Arabidopsis*. Overexpression of *IbZFP1* promotes the activities of NCED (9-cis-epoxy-carotenoid dioxygenase), P5Cs, CAT (catalase), SOD, POD, APX, and in *Arabidopsis*. By Western blot and enzymatic analyses, it was indicated that *IbZFP1* increases the contents of ABA, proline, soluble sugars and chlorophyll and reduces the H_2_O_2_ and MDA (malonaldehyde) contents. Under salt and drought stresses, IbZFP1 could regulate the expression of genes which involved in the process of proline biosynthesis, ROS scavenging, ABA signaling pathway and abiotic stress responses [1]. In general, *IbZFP1* improves salt and drought tolerance by regulating the osmotic substances accumulation and ABA signaling pathway. Zinc finger protein OsZFP213 also enhances tolerance to salt stress by regulating the ABA signaling pathway and scavenging reactive oxygen in rice [68]. *OsZFP213* overexpressing transgenic rice showed more sensitivity to ABA and more salt tolerance compared with *OsZFP213-RNAi* lines and WT in rice. Furthermore, the overexpression of *OsZFP213* could control the expression of antioxidant system genes and the catalytic activity of ROS scavenging enzymes, including SOD, APX, CAT and GR (glutathione reductase), and reduced the accumulation of ROS under salt treatment [68]. In addition, soybean C2H2 transcription factor GmZAT4, including a highly conserved QALGGH motif, induced by stress and ABA, could increase tolerance to PEG and NaCl stress both in soybean and *Arabidopsis* [25].

### 4.2. ABA-Independent Signal Pathway

In the ABA-independent signal pathway, ZFP179 activates the expression of *OsDREB2A* and enhances the ROS-scavenging activity to improve tolerance to oxidative and salt stresses, and differs from the ABA-dependent signal pathway [60]. DST (drought and salt tolerance), also isolated from rice, responds to salt and drought stress via the ABA-independent signal pathway [42]. The expression of *DST* is repressed by drought and salt stresses, resulting in the downregulation of peroxidase 24 precursor, which is related to H_2_O_2_ homeostasis, in turn increasing H_2_O_2_ accumulation, promoting stomatal closure and reducing stomatal density, enhancing tolerance to drought and salt stress in the end. Furthermore, DST was reported to control stomatal closure and responds to salt and drought stresses via ABA-independent instead of the ABA-induced H_2_O_2_ accumulation pathway. In addition, DST could directly bind to the promoter of *LP2* (*plasma membrane receptor-like kinase leaf panicle 2*) and inhibits *LP2* expression to regulate drought sensitivity in rice [69].

ZAT10 (also known as STZ) was first identified as a salt-tolerance-related zinc finger protein, in response to environmental stresses in the ABA-independent signal pathway [70,71,72]. ZAT10 can improve plant tolerance to salt in plants by influencing the expression of ion-balance-related genes and maintaining ionic balance [70,71]. The expression of *ZAT10* in yeast cells compensates the Na^+^ and Li^+^ tolerance of the calcineurin (Ca^2+^/calmodulin-dependent protein) deficient mutant. Consistently, ZAT10 is involved in salt stress response partially dependent on regulating *ENA1* (Na^+^- exporting P-type ATPase gene) or *PMR2* (Ca^2+^ ATPase gene) by the ABA-independent pathway in plants. Similar to AZF, ZAT10 also could recognize two canonical A[G/C]T sequences and contains a conserved EAR motif, which enables ZAT10 to regulate plant tolerance to abiotic stress [33,40]. Many studies have revealed that the EAR motif confers ZAT10 to repress the transcription of different reporter genes in vivo. It was reported that full-length ZAT10 and its repressor domain, including the EAR motif, represses the transcription of *AtERF5* (a class I ERF protein) and *RD29A* (a classical stress-response gene) through binding to their promoter during stress [73]. In addition, *ZAT10* possesses cis-elements in their promoter regions, is strongly induced by various abiotic stresses, such as drought, salt, cold, osmotic stresses and high light [33]. As described above, C2H2-type zinc finger proteins can improve tolerance to abiotic stress by the ROS scavenging system. Under high-light stress, *ZAT10* overexpressing transgenic plants significantly upregulate the expression of the ROS scavenging enzymes, including *APX1*, *APX2*, and *FeSOD1* (*Fe-superoxide dismutase1*), while *ZAT10* knockout plants significantly suppress their expression [70]. Those results suggest that ZAT10 positively regulates high light tolerance by controlling the expression of scavenging ROS-related genes. Interestingly, ZAT10 was reported as possibly acting as both a positive and a negative regulator in response to abiotic stresses. As a positive regulator, overexpressed *ZAT10* in *zat10* mutant lines could rescue tolerance to osmotic stress [70]. However, both the overexpression of *ZAT10* and *ZAT10* knockout or *RNAi* showed more tolerance to osmotic and salinity stresses. ZAT10 might directly activate or repress stress-response genes under abiotic stresses. Under cold stress conditions, ZAT10 could repress the expression of *RD29A* (a classical COR gene), which is a downstream gene of the *CBF3* (*C-repeat binding factor 3*) [73,74,75]. The overexpression of *CBF 3* could increase the expression of *ZAT10*, while a decrease in *CBF3* expression in the *ice1* (*INDUCER OF CBF EXPRESSION 1*) mutant decreases the expression of *ZAT10* in response to cold [75]. CBF3 plays a role in the cold stress response by upregulating the expression of *COR* genes. These studies suggest that ZAT10 might act downstream of *CBF3* and negatively regulate *COR* genes during the cold condition.

Just like ZAT10, ZAT12, which also does not respond to ABA treatment, is also involved in regulating tolerance to multiple abiotic stress, including cold stress, salinity, high light, oxidative stress and osmotic stress [76,77,78,79]. In response to cold stress, more than 20 cold-related genes are upregulated in the *ZAT12* overexpressing transgenic plants [78]. Several studies have shown that *ZAT12* negatively regulates the expression of *CBFs* (*cold-stress-response transcription factors*), such as *CBF1*, *CBF2* and *CBF3*, which may be regulators of *ZAT10* [78]. These results suggest that *ZAT12* acts upstream of *ZAT10* in response to cold stress. In addition, ZAT10 and ZAT12 showed similar expression and response patterns during different abiotic stresses. It is reported that ZAT12 is also involved in the ROS scavenging signal transduction pathway and regulates the expression of *APX1*/*2* and *FSD1*, which are involved in the response to salt and antioxidant stresses [76]. These findings suggest that ZAT10 and ZAT12 function in a coordinated way in response to different stresses.

In addition, *AZF1* and *AZF3* are homologues to *AZF2* and involved in the abiotic stress response in an ABA-independent manner. In contrast to *AZF2*, *AZF1* and *AZF3* showed only a slight response to ABA treatment [33]. *AZF1* and *AZF3* are strongly induced by low temperature and salt stress and improve salt tolerance by controlling downstream ENA1-like genes [33].

### 4.3. ABA-Dependent Signal Pathway

ZAT12, as an active repressor, is involved in abiotic stress regulation via both the ABA-independent signal pathway and the MAPK signaling transduction pathway [76,77,78,79,80,81,82,83]. EIN3 (ethylene-induced 3) phosphorylated by MPK3/6 could directly upregulate the expression of *ZAT12*, which suggested that ZAT12 may be involved in the MAPK signal pathway [84,85,86]. Under Fe deficiency stress, EIN3 plays a positive role in iron Fe uptake, which may be regulated by ROS [85]. H_2_O_2_ level acts as a Fe deficiency response signal, is linked with the regulator FIT (fer-like iron deficiency-induced transcription factor). *FIT* upregulates target genes, such as *FRO2* (*FERRIC REDUCTASE-OXIDASE 2*) and *IRT1* (*IRON-REGULATED TRANSPORTER 1*), in turn, to play a key role in response to Fe deficiency [84]. The FIT could directly interact with EIN3 and EIL1 (EIN3-LIKE 1) and also interact with ZAT12 through its EAR motif [81,83]. Those results suggest that ZAT12 with FIT and EIN3 forms a multiple cross-link network involved in Fe deficiency stress signaling. Under mechanical wounding, ROS and Ca^2+^ connect with MPK8 to play an important role in the recognition and transduction of wound signaling [87]. MPK8 phosphorylated and activated by MKK3 negatively regulates the expression of *ZAT12* by directly inhibiting the *RbohD* gene expression during mechanical wounding [87]. In addition, ZAT12 was essential for the expression of *APX1* and *ZAT7*, which was first identified in the oxidative stress response [76]. During the oxidative stress response, the expression of *ZAT12* and *ZAT7* increased earlier than that of *APX1*. The increased expression of *ZAT12* and *ZAT7* occurs in the loss-of-function mutant *apx1* with increasing H_2_O_2_ levels. ZAT7 was reported to interact with miRNA transport proteins HASATY and WRKY70, and it increases tolerance to salinity stress depending on its EAR domain in *Arabidopsis* [39]. Interestingly, the expression of *ZAT7* without complete EAR domain in transgenic plants does not influence abiotic stress tolerance, while *ZAT7* overexpressing transgenic plants enhance the tolerance to cold stress and decrease the tolerance to osmotic stress, simultaneously [39]. It is suggested that ZAT7 possibly interacts with different factors in different abiotic stress signal pathways. The abovementioned ZAT10 acting downstream of ZAT12 could directly interact with MPKs (MPK3 and MPK6) in vivo and in vitro, which is important for the biological function of ZAT10 in osmotic stress tolerance instead of its EAR motif. A recent report indicates that ZAT10 is directly phosphorylated and regulated by the activated MPKs, and in turn to change the expression of its target genes, finally lead to regulate tolerance to osmotic stress [71]. Those reports indicate that ZAT12, ZAT7 and ZAT10 form a complex regulatory network via the MAPK signaling transduction pathway.

ZAT6, also phosphorylated by MPKs, is strongly activated by drought, cold, Cd (cadmium), salt and osmotic stress [88,89,90,91]. In *Arabidopsis*, *ZAT6*-overexpressing transgenic lines showed to improve the plant’s tolerance to salt stress via the effects of the MAPK cascade on *ZAT6*, but *ZAT6* knockdown lines exhibited decreased stress tolerance [43]. MPKs have been shown to play a central role in response to salt and osmotic stresses, especially MPK6. The activated MPK6 can rapidly phosphorylate the Na^+^/H^+^ antiporter SOS1 (salt overly sensitive) and induce a sodium efflux in the early stages of sodium detoxification [47]. In the longer term, MPK6 directly phosphorylates ZAT6 to regulate the transcriptional transactivation of osmotic- and salt-responsive genes [88]. A recent study found that *ZAT6* positively regulates the plant’s tolerance to Cd stress through controlling the expression of Cd-tolerance-related genes, such as *GSH1* (glutathione*1*), *GSH2*, *PCS1* (phytochelatin synthases) and *PCS2* [92]. In particular, ZAT6 could regulate the expression of *GSH1* through binding to the TACAAT box in its promoter, suggesting that *GSH1* might be the direct target of ZAT6 [92]. In addition, the expression of *CBF1–3* can be directly activated by *ZAT6* binding to the TACAAT domain in its promoter in response to cold [43]. Further research has shown that *ZAT6* is induced by melatonin, which can increase response to cold stress, and subsequently activate the expression of *CBFs* [88]. Interestingly, *CBFs* are also regulated by ZAT12, and function upstream of ZAT10 and AZF1/2/3 via the MAPK signaling transduction pathway. Thus, different ZAT proteins may be highly related and involved in response to abiotic stress in a similar, coordinated and cooperative manner.

In the ABA signaling pathway, the expression level of *ZFP36* is increased by ABA-triggered H_2_O_2_ production and the MAPKs activity in response to water stress and oxidative stress [64]. Meanwhile, ZFP36 also regulates the expression of NADPH oxidase and MAPK genes and the production of H_2_O_2_ in ABA signaling [64]. These reports suggest that ZFP36 interacting with MAPK and ABA signaling, NADPH oxidase and H_2_O_2_ form the crosstalk for the regulation of tolerance to water stress and oxidative stress in rice. On the other hand, OsZFP213 also interacts directly with OsMAPK3 to regulate salt tolerance by increasing the ability of ROS scavenging [68]. Moreover, the interaction between OsZFP213 and OsMAPK3 could improve the transactivation activity of OsZFP213 [68].

## 5. Conclusions

C2H2 zinc finger proteins have been widely reported for their response to a wide spectrum of abiotic stress, such as drought, extreme temperatures, salinity, excessive light, osmotic and oxidative stress (Table 1) [1,5]. Among them, the C1-2i Q-type C2H2 zinc finger proteins subclass was the most extensively studied to be associated with abiotic stress responses in plants. The C1-2i subclass contains 20 members in Arabidopsis, and many of them have been reported to play crucial roles in abiotic stresses response, such as ZAT6, ZAT7, ZAT10, ZAT12 and AZF1/2/3 [2,7]. Previous studies imply that different C2H2 zinc finger proteins display some similarities and distinctions in regulating abiotic stress tolerance in plants [2,5,22,25]. Most members of C2H2 zinc finger proteins involved in the abiotic stresses response, such as ZFP245, ZFP179, ZAT7, ZAT10, ZAT12 and AZF1/2/3, function as repressors through the EAR motif. ZAT10 and AZF1/2/3 can recognize the same A[G/C]T core sequences of A (G/C) T-X3~4-A (G/C) T [33,39,60,63,70,76]. There are four key characteristics that are exhibited in C2H2-type zinc finger proteins participating in the abiotic stress response. Firstly, C2H2 zinc finger proteins play a multifunctional role in stress resistance and improve plant stress tolerance via various pathways at the same time. The C2H2 zinc finger protein can regulate the plant response to abiotic stress by interacting with stress response factors, be directly induced by abiotic stress, influencing stress-related plant phenotype, and cross talk with phytohormone [1,2,5,8]. For example, ZFP36 regulates the stress-related genes and influences NADPH oxidase and H_2_O_2_ activity and is also involved in the MAPK and ABA signaling pathways in rice [64]. Secondly, the function of C2H2 zinc finger protein is versatility. C2H2 zinc finger protein can participate in multiple stress regulatory pathways, and even integrate different stress regulatory pathways. PtrZPT2-1, a ZPT2 family transcription factor from trifoliate orange, can increase plant tolerance to low temperature, drought and salinity in tobacco [93]. ZFP5 regulates root hair development by integrating the phosphate and potassium stress by ethylene signaling in *Arabidopsis* [94]. Thirdly, C2H2 zinc finger proteins have similar or opposite functions in regulating plant stress resistance. ZAT10, functioning as both a positive and a negative regulator, is involved in response to various environmental factors, including drought, salinity, heat and osmotic stress in various plant species [2,5,22]. Fourthly, C2H2 zinc finger proteins have cooperative and complementary or overlapping roles in the signal transduction pathway under abiotic stresses. In the ABA signaling pathway, *SCOF-1* enhances cold tolerance by interacting with *SGBF-1* (a bZIP transcription factor) to promote the expression of *COR* [5,55,56,57]. ZAT10 could repress the expression of *RD29A* (a classical COR gene) which is a downstream gene of the *CBF3* (*C-repeat binding factor 3*). *ZAT12* negatively regulates the expression of *CBF3* and is also regulated by ZAT12 and *ZAT6* in response to cold stress via the MAPK signaling transduction pathway [43,47,78]. Thus, it is very important to screen the proteins interacting with C2H2 zinc finger proteins and identify the upstream and downstream of C2H2 zinc finger protein genes and elucidate the complex network and molecular mechanism of plant responses to abiotic stresses via the C2H2 zinc finger protein pathway.

Recently, many C2H2-type zinc finger proteins were reported and studied not only in model plants (such as *Arabidopsis*, cotton, wheat, soybean and rice), but also in economic crops (tomato, cucumber, potato, apple and tobacco) [22,23,24,25,27,28,96,97]. For example, OsDRZ1 (drought-responsive zinc finger protein 1) positively regulates stress tolerance through the ROS scavenging system in *Oryza sativa* [95]. *OsZFP350* improves tolerance to abiotic stress and increases the adaptability of *Oryza sativa* roots [23]. MdZAT10, a homolog of Arabidopsis ZAT10, induced by abiotic stress and ABA treatments, plays a negative role in the drought resistance in apple [22]. A variety of research methods were applied to the study of C2H2 zinc finger protein, which is involved in the abiotic stress response. However, there are still many issues that need to be solved, such as the corresponding relation between the structure and function of C2H2-zinc finger proteins, the detail of C2H2 zinc finger protein capturing the stress signal, the distribution of C2H2 zinc finger proteins in the different pathways, and the regulation mechanisms of complex C2H2 zinc finger protein network in the abiotic stress response. Furthermore, abiotic stress factors influence plant growth and development, and finally influence crop quality and yield in agricultural production [5]. Therefore, more studies should focus on how to apply the theory of C2H2 zinc finger protein in response to abiotic stress for molecular crop breeding. We now recognize the enormous significance of the C2H2 zinc finger proteins research in abiotic stress response and need to make great efforts to increase plant stress tolerance and improve crop yield via using C2H2 zinc finger proteins for crop molecular breeding in the future.

## Figures and Tables

**Table 1:** C2H2 zinc finger proteins from different plant species response to various abiotic stresses.

C2H2 Zinc Finger Protein	Responsive to Abiotic Stress	Plants	References
SCOF-1	Low temperature stress	*Gycin emax, Solamum tuberosum*	[55,56,57]
ZFP245	Cold, drought and oxidative stress	*Oryza sativa*	[58,59]
ZFP179	Salt stress	*Oryza sativa*	[60,61]
AZF2	Cold, drought and salt stress	*Arabidopsis thaliana*	[33,62]
ZFP36	Drought and oxidative stress	*Oryza sativa*	[64,65,66]
IbZFP1	Salt and drought stress	*Ipomoea batatas*	[67]
OsZFP213	Salt stress	*Oryza sativa*	[68]
GmZAT4	Salt and drought stress	soybean, *Arabidopsis thaliana*	[25]
DST	Salt and drought stress	*Oryza sativa*	[42,69]
ZAT10/STZ	Drought, high-light, salt, cold, oxidative stress and osmotic stress	*Arabidopsis thaliana*	[22,70,71,72]
ZAT12	Drought, high-light, salt, cold, oxidative stress and osmotic stress	*Arabidopsis thaliana*	[76,77,78,79,80,81,82,83]
AZF1, 3	Salt and cold stress	*Arabidopsis thaliana*	[33,63]
ZAT7	Salt, cold and oxidative stress	*Arabidopsis thaliana*	[39]
ZAT6	Drought, cold, Cd, salt and osmotic stress	*Arabidopsis thaliana*	[88,89,90,91,92]
ZPT2-1	Cold, drought and salt stress	*Petunia hybrida tobacco*	[93]
ZFP5	phosphate and potassium stress	*Arabidopsis thaliana*	[94]
OsDRZ1	Drought stress	*Oryza sativa*	[95]
OsZFP350	Heat, salinity and drought stress	*Oryza sativa*	[23]
MdZAT10	Drought stress	Apple	[22]

## Data Availability

Not applicable.
